# Supplementary material for: Clinical severity of SARS-CoV-2 Omicron BA.4 and BA.5 lineages compared to BA.1 and Delta in South Africa
Source: Nat Commun. 2022 Oct 4;13:5860. doi: 10.1038/s41467-022-33614-0 (PMC9531215; doi:10.1038/s41467-022-33614-0)
Supplement: Supplementary file 1 — Supplementary Information [file 41467_2022_33614_MOESM1_ESM.pdf]

SUPPLEMENTARY MATERIAL

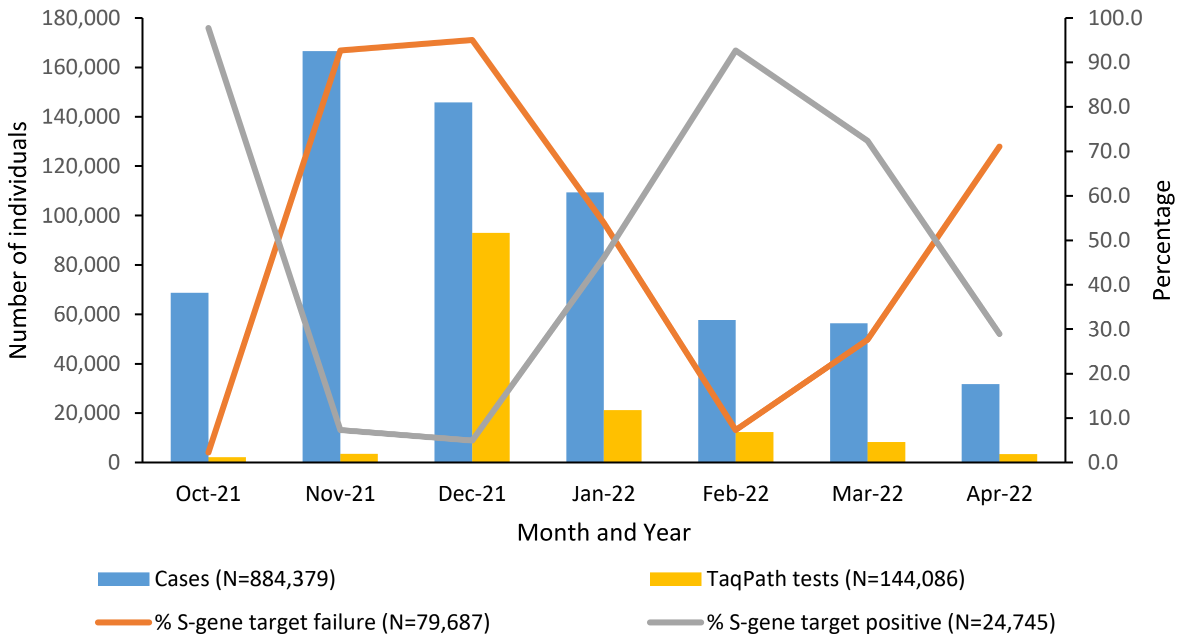

Supplementary Figure 1. Number of cases detected, infections diagnosed using the TaqPath test, and percentage of S-gene target positive (SGTP) and S-gene target failure (SGTF) infections by month, 1 October 2021 – 26 April 2022

Supplementary Table 1. Percentage of SARS-CoV-2 variant/lineage based on genomic surveillance data from NGS-SA[1], and SGTF and SGTP based on TaqPath PCR data by month of diagnosis, South Africa, October 2021 – April 2022

	Genomic surveillance data[1]					TaqPath PCR data	
	**Delta (%)**	**BA.1 (%)**	**BA.2 (%)**	**BA.4/BA.5 (%)**	**Other (%)**	**SGTF (%)**	**SGTP (%)**
October 2021	86.0	0.5	0.0	0.0	13.5	2.3	97.2
November 2021	10.1	86.9	0.4	0.0	2.8	92.7	7.3
December 2021	0.3	92.9	5.0	0.0	1.8	95.1	4.9
January 2022	0..3	55.1	42.9	0.1	1.5	54.0	46.0
February 2022	0.5	11.8	85.8	0.5	1.4	7.3	92.7
March 2022	0.6	4.2	77.6	16.2	1.4	27.6	72.4
April 2022	0.1	1.3	25.1	73.0	0.5	71.1	28.9

SGTP: S-gene target positive, SGTF: S-gene target failure

Blue highlighting: SGTP infections diagnosed in October and November 2021 were classified as Delta, SGTF infections diagnosed between November 2021 through January 2022 were classified as BA.1, SGTP infections diagnosed from February through April 2022 were classified as BA.2 and SGTF infections diagnosed in April 2022 were classified as BA.4/BA.5.

Supplementary Table 2. Number of SARS-CoV-2 TaqPath PCR detected infections, incidence and hospitalisations by province, October 2021 – April 2022

Province	TaqPath PCR-detected infections[a] n/N (%)	Incidence per 100,000 persons	No. of hospitalisations
**Eastern Cape**	2193/60097 (3.7)	866.0	6626
**Free State**	3462/43631 (7.9)	1433.4	5408
**Gauteng**	74983/317340 (23.6)	2050.5	26206
**KwaZulu-Natal**	29437/148263 (19.9)	1437.9	16307
**Limpopo**	4401/35531 (12.4)	573.9	3666
**Mpumalanga**	9582/45358 (21.1)	909.7	4101
**North West**	6655/44148 (15.1)	1038.5	4826
**Northern Cape**	1916/19315 (9.9)	1433.3	1870
**Western Cape**	8768/155535 (5.6)	2112.0	19151
Unknown	2689/15161 (17.7)	0	0
**Total**	144086/884379 (16.3)	1460.7	88161

[a] For the period 1 October 2021 – 26 April 2022
