# Supplementary material for: Clinical severity of SARS-CoV-2 Omicron BA.4 and BA.5 lineages compared to BA.1 and Delta in South Africa
Source: Nat Commun. 2022 Oct 4;13:5860. doi: 10.1038/s41467-022-33614-0 (PMC9531215; doi:10.1038/s41467-022-33614-0)
Supplement: Supplementary file 3 — Reporting Summary [file 41467_2022_33614_MOESM3_ESM.pdf]

nature portfolio

Corresponding author(s): Nicole Wolter

Last updated by author(s): Sep 19, 2022

Reporting Summary

Nature Portfolio wishes to improve the reproducibility of the work that we publish. This form provides structure for consistency and transparency in reporting. For further information on Nature Portfolio policies, see our Editorial Policies and the Editorial Policy Checklist.

Statistics

For all statistical analyses, confirm that the following items are present in the figure legend, table legend, main text, or Methods section.

n/a	Confirmed	
☐	☒	The exact sample size (*n*) for each experimental group/condition, given as a discrete number and unit of measurement
☒	☐	A statement on whether measurements were taken from distinct samples or whether the same sample was measured repeatedly
☐	☒	The statistical test(s) used AND whether they are one- or two-sided<br>*Only common tests should be described solely by name; describe more complex techniques in the Methods section.*
☐	☒	A description of all covariates tested
☒	☐	A description of any assumptions or corrections, such as tests of normality and adjustment for multiple comparisons
☐	☒	A full description of the statistical parameters including central tendency (e.g. means) or other basic estimates (e.g. regression coefficient) AND variation (e.g. standard deviation) or associated estimates of uncertainty (e.g. confidence intervals)
☐	☒	For null hypothesis testing, the test statistic (e.g. *F*, *t*, *r*) with confidence intervals, effect sizes, degrees of freedom and *P* value noted<br>*Give P values as exact values whenever suitable.*
☒	☐	For Bayesian analysis, information on the choice of priors and Markov chain Monte Carlo settings
☒	☐	For hierarchical and complex designs, identification of the appropriate level for tests and full reporting of outcomes
☒	☐	Estimates of effect sizes (e.g. Cohen's *d*, Pearson's *r*), indicating how they were calculated

Our web collection on statistics for biologists contains articles on many of the points above.

Software and code

Policy information about availability of computer code

Data collection	DATCOV data were submitted through an electronic (web-based) platform and stored in a Microsoft Azure SQL database. The national COVID-19 case database is a laboratory-based surveillance programme which receives real-time electronic data on all laboratory-confirmed SARS-CoV-2 cases in South Africa through the Notifiable Medical Conditions (NMC) system.
Data analysis	Analysis was performed using Stata 14.1® (StataCorp LP, College Station, US). The code generated and used during the current study have been deposited and are available in a public repository (https://doi.org/10.5281/zenodo.7085507#).

For manuscripts utilizing custom algorithms or software that are central to the research but not yet described in published literature, software must be made available to editors and reviewers. We strongly encourage code deposition in a community repository (e.g. GitHub). See the Nature Portfolio guidelines for submitting code & software for further information.

Data

Policy information about availability of data

All manuscripts must include a data availability statement. This statement should provide the following information, where applicable:

- Accession codes, unique identifiers, or web links for publicly available datasets
- A description of any restrictions on data availability
- For clinical datasets or third party data, please ensure that the statement adheres to our policy

The data generated and analysed during the current study contain potentially identifiable information and were shared with the national public health institute

under the Notifiable Medical Conditions (NMC) regulations and therefore have restricted access due to privacy and ethical issues. Access to aggregated data can be obtained by request to the corresponding author, Nicole Wolter (nicolew@nicd.ac.za), and will be subject to proof of an IRB-approved protocol and signature of a data sharing agreement. Responses to requests will be within three weeks from request receipt.

Human research participants

Policy information about studies involving human research participants and Sex and Gender in Research.

Reporting on sex and gender	Only sex was considered in the study design and analyses.
Population characteristics	The median age and interquartile range of cases with known variant/lineage was 37 (26-49) years. Females accounted for 55.7% (54,450/97,726) of cases.
Recruitment	This is a secondary data analysis, linking multiple existing datasets.
Ethics oversight	Human Research Ethics Committee (Medical) of University of the Witwatersrand

Note that full information on the approval of the study protocol must also be provided in the manuscript.

Field-specific reporting

Please select the one below that is the best fit for your research. If you are not sure, read the appropriate sections before making your selection.

☒ Life sciences ☐ Behavioural & social sciences ☐ Ecological, evolutionary & environmental sciences

For a reference copy of the document with all sections, see nature.com/documents/nr-reporting-summary-flat.pdf

Life sciences study design

All studies must disclose on these points even when the disclosure is negative.

Sample size	We conducted a retrospective secondary data analysis of existing datasets. Sample size was not calculated as we used all data available at the time of the analysis.
Data exclusions	No data exclusions
Replication	Not applicable - this was not an experimental study. We conducted a secondary data analysis of data from the national COVID-19 case surveillance programme.
Randomization	Not applicable - this was an observational study and there was no intervention in the study design.
Blinding	Not applicable - this was an observational study and there was no intervention in the study design.

Reporting for specific materials, systems and methods

We require information from authors about some types of materials, experimental systems and methods used in many studies. Here, indicate whether each material, system or method listed is relevant to your study. If you are not sure if a list item applies to your research, read the appropriate section before selecting a response.

Materials & experimental systems

n/a	Involved in the study	
☒	☐	Antibodies
☒	☐	Eukaryotic cell lines
☒	☐	Palaeontology and archaeology
☒	☐	Animals and other organisms
☒	☐	Clinical data
☒	☐	Dual use research of concern

Methods

n/a	Involved in the study	
☒	☐	ChIP-seq
☒	☐	Flow cytometry
☒	☐	MRI-based neuroimaging
